# Association Between Sleep Duration and Angina Characteristics in United States Adults

**DOI:** 10.1016/j.ajmo.2025.100109

**Published:** 2025-06-19

**Authors:** Maslahuddin HA Alhaque Roomi, Nehal Eid, Aayush Visaria

**Affiliations:** a Mercy Catholic Medical Center, Pennsylvania, USA; b Mansoura Manchester Medical Program, El Mansoura, Egypt; c Rutgers Robert Wood Johnson Medical School, New Jersey, USA

**Keywords:** Sleep duration, Angina, Cross-Sectional, NHANES

## Abstract

**Background:**

Sleep is now recognized as a key factor in cardiovascular health by the American Heart Association's Life’s Essential 8. However, the relationship between sleep duration and stable angina remains unexplored.

**Methods:**

This nationally representative cross-sectional study analyzed data from 18,385 U.S. adults aged 40 and older using the National Health and Nutrition Examination Survey (2005-2018). Daily sleep duration was categorized as <7 hours, 7-8 hours (reference), and >8 hours. Angina was assessed with the Rose Angina Questionnaire and classified by severity (Grade 1 or 2) and pain location (typical vs atypical). Covariates were identified a priori based on previous literature, and clinical relevance.

**Results:**

Our study included 18,385 adults with a mean age of 57.6 years (SE 0.16). Out of these, 48.6% were female and 70% were non-Hispanic Whites. A total of 954 (5.2 %) participants reported experiencing angina. Among those with angina, 109 (11%) reported atypical symptoms. Univariate analysis revealed that both short (<7 hours) and long (>8 hours) sleep durations were associated with higher odds of Grade 2 angina compared to adequate sleep (7-8 hours). Adjusted analysis showed significantly higher odds of Grade 2 angina in individuals sleeping >8 hours (OR [95% CI]: 2.16 [1.08-4.32] for females; 2.69 [1.15-6.29] for males). Additionally, sleep <7 hours was associated with a greater likelihood of atypical angina presentation (OR: 1.77 [1.21-3.05]).

**Conclusion:**

Our findings suggest that sleeping over 8 hours increases the likelihood of Grade 2 angina, while under 7 hours is linked to atypical presentations, complicating diagnosis. Clinicians could incorporate brief sleep assessments—asking about duration and quality—alongside angina tools like the ROSE questionnaire to identify potential sleep-related factors. While promising, these associations require further research before being translated into definitive clinical guidelines for angina management.

## 1 Introduction

Sleep has recently been included in the American Heart Association’s (AHA) Life’s Essential 8, emphasizing its crucial, yet underappreciated role, in driving cardiovascular risk. It quietly accounts for about 30% of our existence,^1^ and is linked to cardiovascular health (coronary artery disease, heart failure, arrhythmias, and diabetes^2^^,^^3^), immune system, hormone balance, cognition, and memory.^4^ Unfortunately, 10% to 30% of US adults struggle with sub-optimal sleep and insomnia,^5^ while over 50% of those aged 65 and above experience sleep disorders.^6^

There is a lack of data on the association between sleep duration and stable angina, which is imperative to understand as inflammatory^7^, ^8^, ^9^ and stress responses change with sleep and may affect both presence of angina and angina characteristics (eg, location, severity of pain). We sought to understand the association between sleep duration and angina in a nationally representative U.S. adult population.

## 2 Materials and Methods

### 2.1 Study Population

We conducted a cross-sectional study utilizing data from the 2005-2018 cycles of the National Health and Nutrition Examination Survey (NHANES). NHANES, managed by the National Center for Health Statistics (NCHS) at the Centers for Disease Control and Prevention (CDC), provides comprehensive national health statistics through interviews, medical examinations, and laboratory analyses of a representative sample of the U.S. population.

**Figure 1:**
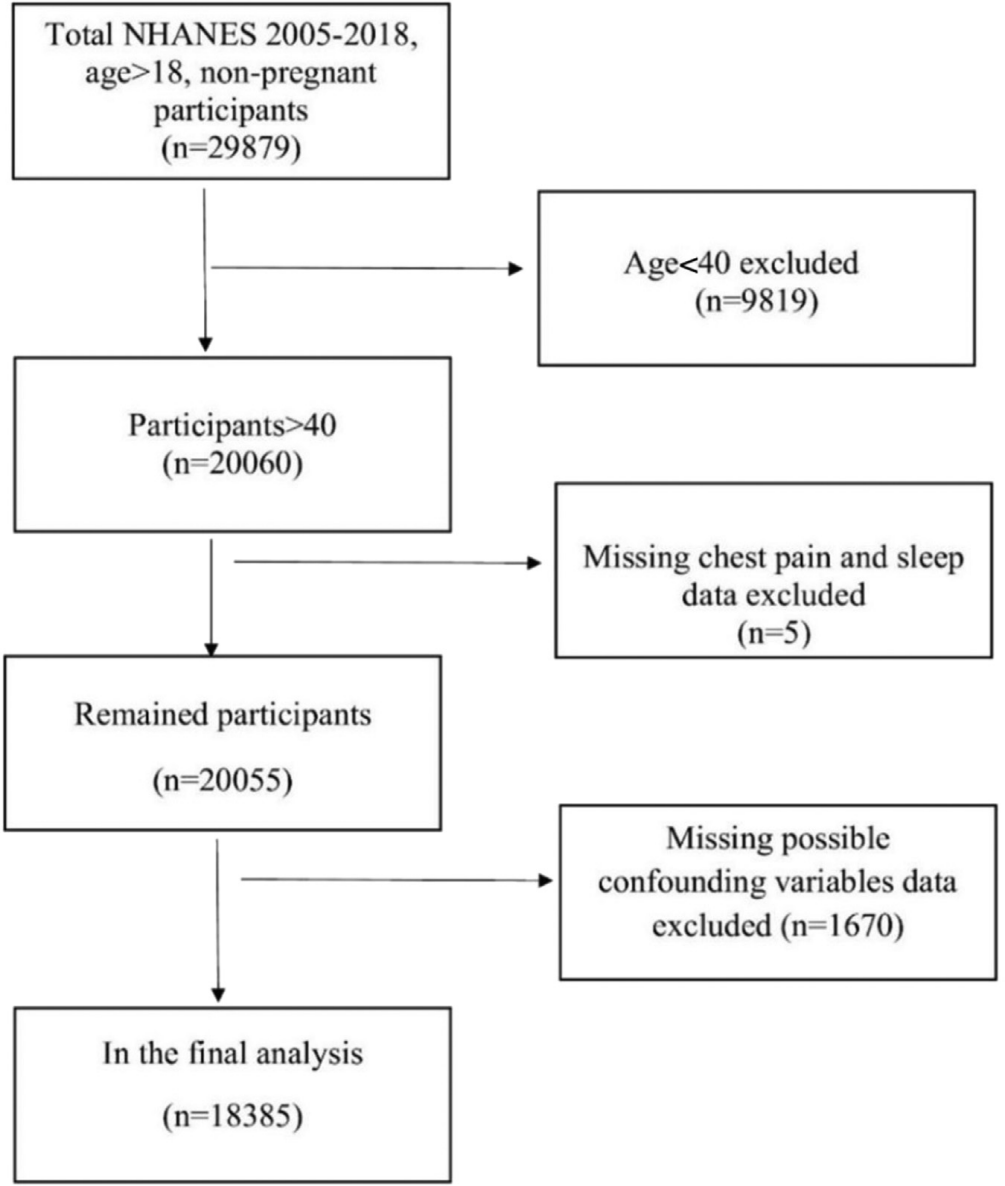
Flowchart of participant selection. A total of 18,385 adults aged 40 years and older were included in the final analysis. Participants younger than 40 years (n = 9819), those with incomplete chest pain or sleep data (n = 5), and individuals with missing information on key confounding variables (n = 1670) were excluded.

We included 18,385 adult participants aged 40 years and older, after excluding participants younger than 40 years (n = 9819), those with incomplete chest pain or sleep data (n = 5), and with missing data on potential confounding variables (n = 1670) were excluded from the analysis (Figure 1).

Given that NHANES is a publicly available dataset, our research was not subject to human subject research regulations per Rutgers Institutional Review Board regulations. However, it is important to note that NHANES requires informed consent from participants and maintains the confidentiality of their personal information.

### 2.2 Exposure and Outcome Variables

Sleep duration was evaluated using a self reported questionnaire asking participants “How much sleep do you usually get at night on weekends or workdays?” ^10^ or “Number of hours usually slept on weekdays or workdays.” and “Number of hours usually slept on weekends or nonworkdays.”^11^ Based on recommended daily sleep hours for adults,^12^ we classified sleep duration into 3 categories; ≤7 hours, 7-8 hours (reference),^12^ and > 8 hours. Angina was evaluated using a validated Rose Angina Questionnaire (RAQ).^13^ RAQ classified patients with angina into two grades according to the presence of pain at ordinary pace: Grade 1 (absence of pain during ordinary pace on level ground) or Grade 2 (presence of pain during ordinary pace on level ground). The majority of patients with cardiac angina had left sided or sternal chest pain. Therefore, we additionally classified participants into two groups according to the site of pain: typical (left sided or sternal pain) or atypical (right sided, neck or epigastric pain).

Our primary outcome is the presence of Grade 1 (yes/no) and Grade 2 angina (yes/no) and secondary outcomes include the location of pain in these groups.

### 2.3 Covariates

Covariates included demographic factors (age, sex, race, education level [high school graduate or higher], smoking status [current, former, never], and poverty index [income-to-poverty ratio]) and clinical variables. Clinical covariates encompassed mean systolic blood pressure, impaired glucose tolerance (defined by self-reported diabetes, antidiabetic medication use, fasting blood glucose ≥100 mg/dL, or HbA1C ≥5.7%), cancer, COPD, and previous cardiovascular disease (history of stroke, nonfatal myocardial infarction, or heart failure). Anthropometric (BMI, waist circumference) and laboratory measures (direct HDL, triglycerides) were also included. Age, sex, and race were adjusted for due to their role in cardiovascular risk stratification.^25^^,^^26^ Socioeconomic factors influence healthcare access and lifestyle. Smoking, blood pressure, diabetes, and dyslipidemia contribute to atherosclerosis, while obesity-related measures reflect metabolic burden.^25^^,^^26^ Chronic conditions like COPD and cancer impact inflammation and cardiopulmonary function.^27^ Adjusting for these covariates allowed us to isolate the association between sleep duration and angina while minimizing confounding effects.

### 2.4 Statistical Analysis

We divided participants into three groups based on their daily sleep hours at night: <7 hours, 7-8 hours, and >8 hours. Baseline characteristics for continuous variables were presented in the form of mean and standard error, and categorical variables as N (weighted percentage) form. Based on previous literature,^25^, ^26^, ^27^ we adjusted for confounders a priori using multivariable logistic regression equations to examine the relationship between the daily sleep hours and Grade 1 and Grade 2 angina occurrence. The first model had no covariates adjusted; the second model adjusted for all aforementioned covariates. We employed sensitivity analyzes recategorizing sleep duration (<6, 6-9, >9 hours) to determine the robustness of the primary association, data is available as supplementary material*. All analyzes accounted for the complex survey design using poststratification weights and clustering. Statistical analyses were performed using SAS

9.4 (Cary, NC). Statistical significance was defined as *P* < .05.

## 3 Results

### 3.1 Characteristics of the Study Population

Our study included 18,385 adults with a mean age of 57.6 years (SE 0.16). Out of these, 48.6% were female and 70% were non-Hispanic Whites. A total of 954 (5.2 %) participants reported experiencing angina. Among those with angina, 109 (11%) reported atypical symptoms. The baseline characteristics are mentioned in Table 1.

**Table 1:** Baseline Characteristics of Study Population.

Male	48.2%					
Female	48.6%					

	Mean			Standard Error (SE)		

Age	57.6			0.2		
BMI	29.3			0.1		
Waist Circumference	101.5			0.2		
Weight (kg)	83.04			0.3		
Mean SBP	125.8			0.3		
Mean DBP	71.8			0.2		
Pulse	71.5			0.2		
Triglycerides (mg/dL)	160.7			1.4		
Direct HDL (mg/dL)	54.4			0.2		
Glycohemoglobin	5.8			0.01		
Sleep Duration (hours)	7.1			0.01		

Label	Sleep < 7 Hours/Day Mean(SE)		Sleep 7-8 Hours/Day Mean(SE)		Sleep >8 Hours/Day Mean(SE)	
	Male	Female	Male	Female	Male	Female

Age in years at screening	56.5 (0.2)	55.2 (0.3)	57.8 (0.2)	57.1 (0.2)	61.6 (0.4)	62.9 (0.6)
BMI (kg/m2)	30.2 (0.2)	29.5 (0.2)	28.9 (0.2)	29.1 (0.1)	29.7 (0.3)	29.2 (0.2)
Waist Circumference (cm)	99.5 (0.4)	104.7 (0.4)	97.5 (0.4)	104.6 (0.3)	99.8 (0.7)	105.8 (0.6)
Weight (kg)	78.2 (0.5)	90.7 (0.5)	75.4 (0.4)	90.1 (0.4)	76.6 (0.8)	88.6 (0.7)
SBP	125.5 (0.4)	125.7 (0.4)	124.4 (0.4)	126.3 (0.3)	127.9 (0.6)	128.6 (0.6)
DBP	71.2 (0.3)	74.4 (0.3)	70.2 (0.3)	73.2 (0.3)	69.5 (0.5)	71.3 (0.5)
Pulse	73.3 (0.3)	70.7 (0.4)	71.9 (0.2)	70.1 (0.3)	72.9 (0.5)	71.9 (0.6)
Triglycerides (mg/dL)	146.5 (2.2)	179.5 (4)	142.3 (1.8)	176.7 (2.6)	152.2 (2.9)	172.4 (5.3)
HDL-Cholestero l (mg/dL)	58.6 (0.4)	47.5 (0.3)	61.04 (0.4)	48.4 (0.4)	60.4 (0.8)	49.1 (0.7)
Glycohemoglobin (%)	5.8 (0.02)	5.9 (0.03)	5.7 (0.02)	5.8 (0.02)	5.8 (0.03)	5.9 (0.04)
Sleep duration (hours)	5.5 (0.02)	5.6 (0.02)	7.5 (0.01)	7.5 (0.01)	9.3 (0.03)	9.4 (0.03)

BMI = body mass index; SBP = systolic blood pressure; DBP = diastolic blood pressure; HDL = high-density Lipoprotein.

**Figure 2:**
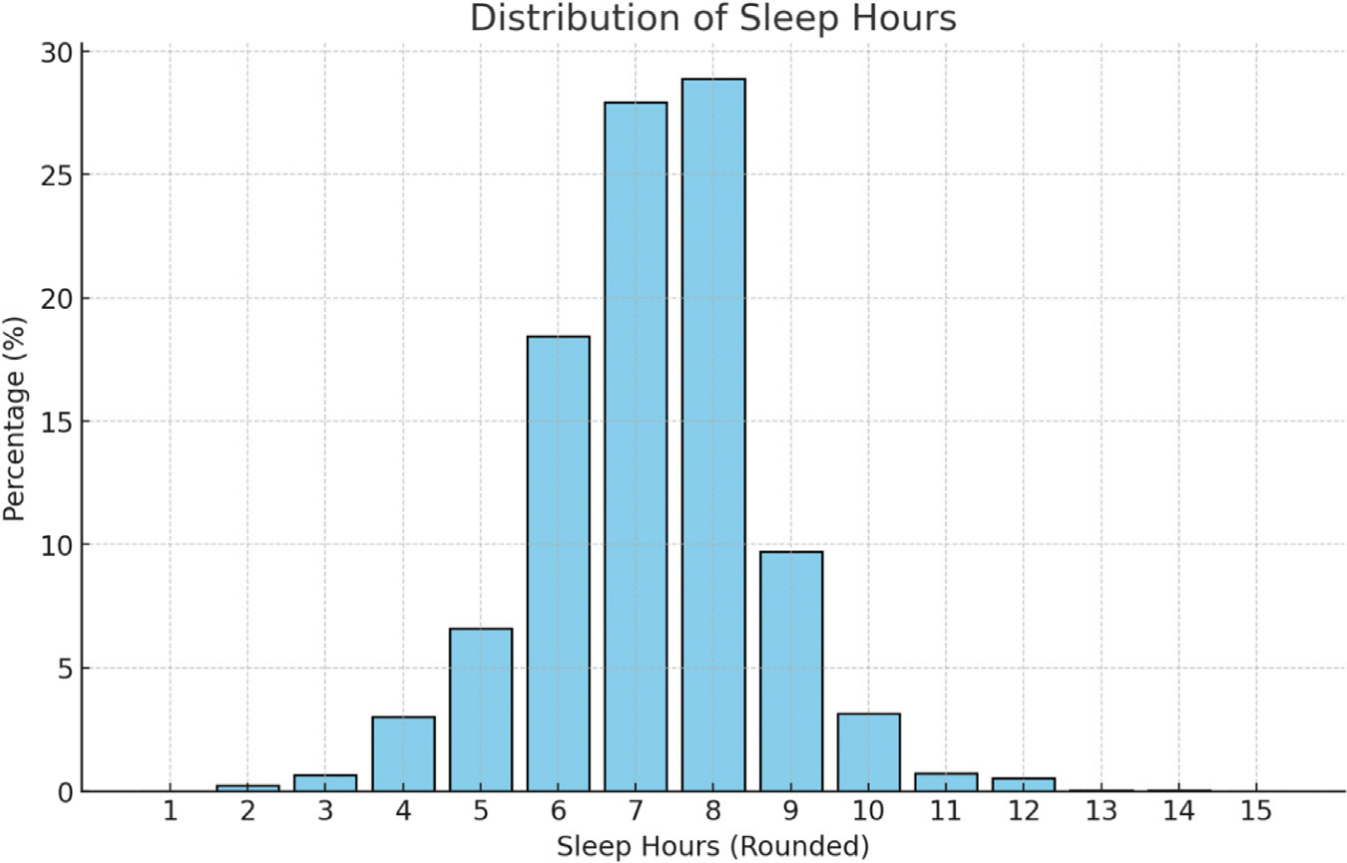
Distribution of sleep duration in the study population. This graph displays the percentage of participants by reported hours of sleep. The distribution follows a bell-shaped curve, with the highest proportion of individuals sleeping 6 to 8 hours per day.

The distribution of sleep hours in the study population is provided in Figure 2.

### 3.2 Univariate Analysis

Univariate analysis (Table 2) revealed that individuals sleeping less than 7 hours or more than 8 hours per day had significantly higher odds of experiencing Grade 2 angina compared to those with 7-8 hours of sleep. Among females, the odds ratios (OR) were 2.11 [1.16-3.86] for <7 hours and 2.69 [1.26-5.78] for >8 hours; among males, 2.39 [1.25-4.55] and 3.84 [1.64-9.01], respectively.

**Table 2:** Odds Ratio of Grade 2 Angina, Grade 1 Angina, and Atypical Angina Pain for Different Daily Sleep Categories as Compared with 7-8 Hours of Daily Sleep.

A. Odds of Grade 2 Angina				
a.	Males	Daily Sleep	OR	95% CI
		<7 Hours	2.39	1.25-4.55*
		>8 Hours	3.84	1.64-9.01*
	Females	<7 Hours	2.11	1.16-3.86*
		>8 Hours	2.69	1.26-5.78*
b.	Combined Males and Females	<7 Hours	3.11	1.85-5.22*
		>8 Hours	2.58	1.53-4.36*

B. Odds of Grade 1 Angina				

a.	Males	<7 Hours	1.23	0.81-1.85
		>8 Hours	1.29	0.85-1.97
	Females	<7 Hours	1.11	0.62-1.98
		>8 Hours	1.12	0.71-1.76
b.	Combined Males and Females	<7 Hours	1.39	1.002-1.939*
		>8 Hours	1.92	1.21-3.05*

C. Atypical Angina				

a.	Both Males and Females	<7 Hours	1.77	1.004-3.116*
		>8 Hours	1.33	0.66-2.69

OR = Odds Ratio, CI = confidence interval.

*Ratio is significant at 0.05 level.

A Odds of Grade 2 Angina for different daily sleep categories as compared with 7-8 hours of daily sleep.

B Odds of Grade 1 Angina for different daily sleep categories as compared with 7-8 hours of daily sleep.

C Odds of Atypical Angina pain for different daily sleep categories as compared with 7-8 hours of daily sleep.

In the overall (nonstratified) model, Grade 2 angina was associated with ORs of 3.11 [1.85-5.22] for <7 hours and 2.58 [1.53-4.36] for >8 hours of sleep. Grade 1 angina was also more likely in those with sleeping <7 hours or >8 hours, with ORs of 1.39 [1.002-1.939] and 1.92 [1.21-3.05], respectively.

Additionally, participants sleeping <7 hours/day had increased odds of reporting atypical chest pain during an acute angina episode (OR: 1.77 [1.004-3.116]).

**Figure 3:**
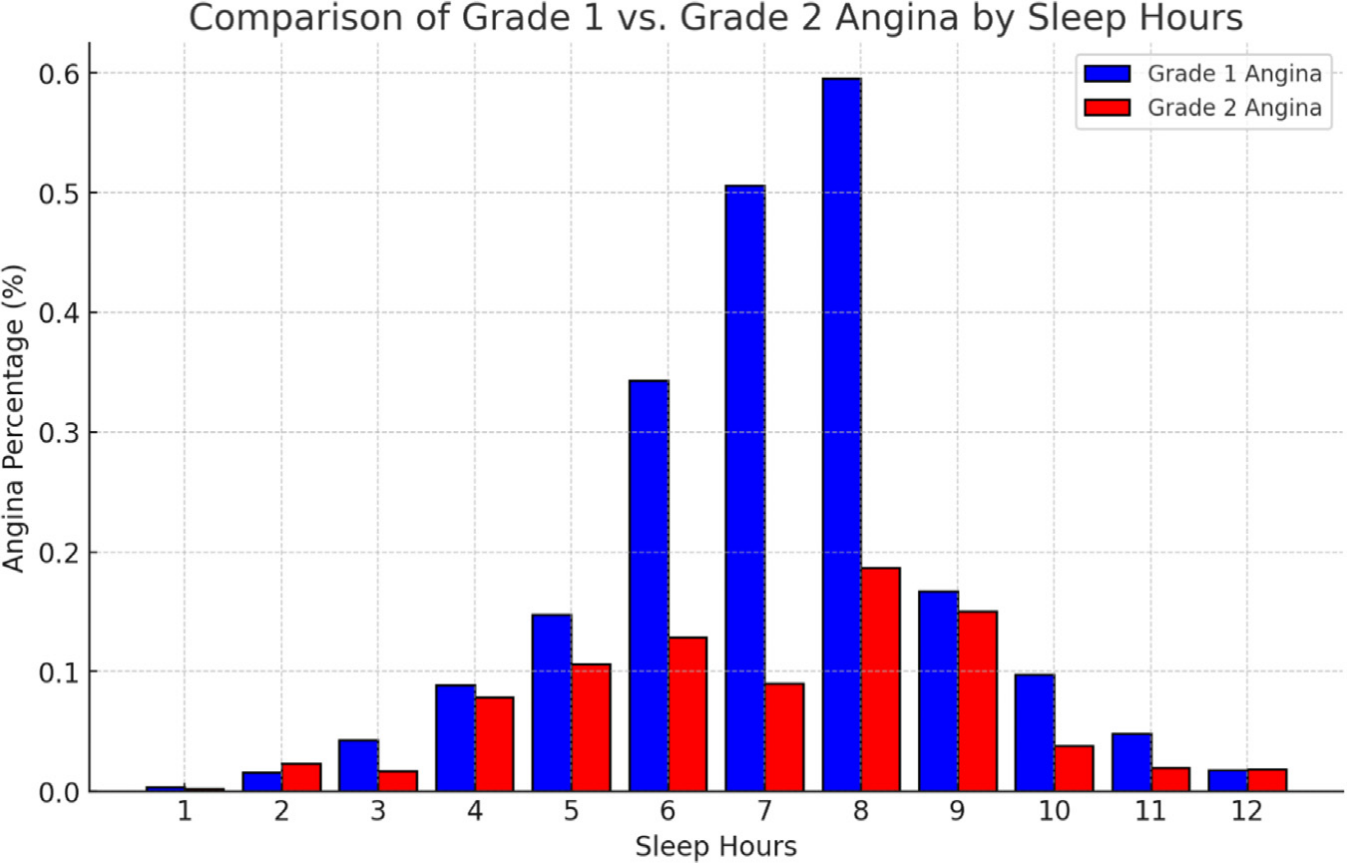
Percentage distribution of angina severity by sleep duration. This bar graph shows the proportion of individuals with Grade 1 (blue) and Grade 2 (red) angina symptoms across different sleep duration categories. The values are shown relative to the total population, highlighting differences in symptom severity with varying sleep durations.

Figure 3 illustrates the percentage distribution of the study population experiencing Grade 1 and Grade 2 Angina, stratified by sleep hours, in relation to the total population with angina.

### 3.3 Adjusted Analysis: Odds of Grade 2 Angina

After adjusting for covariates, the odds of Grade 2 angina continued to remain significantly elevated in participants having daily sleep >8 hours (OR [95% CI]; Female: 2.16 [1.08-4.31]; Male: 2.69 [1.15-6.29]). However, no such difference was observed for individuals getting <7 hours of daily sleep (Female 1.67 [0.92-3.05]; Male 1.80 [0.92-3.54]).

Table 3

**Table 3:** Adjusted Odds Ratios for Grade 2 Angina: Females vs. Males.

Adjusted Odds Ratio Estimates				
	Female		Male	
Effect	Point Estimate	95% CI	Point Estimate	95% CI
Sleep <7 hours/ Day†	1.67	0.92-3.05	1.8	0.92-3.54
Sleep >8 hours/ day†	2.16	1.08-4.31⁎	2.69	1.15-6.29⁎
Covariates				
Age	0.98	0.96-0.99*	0.99	0.97-1.03
Mexican American‡	1.59	0.73-3.47	0.68	0.27-1.68
Other Hispanic‡	2.87	1.44-5.73*	2.08	0.81-5.39
Non-Hispanic Black‡	1.9	0.99-3.63	1.32	0.65-2.71
Other race—including multiracial‡	2.24	0.78-6.40	0.63	0.21-1.87
Alcohol consumption	0.94	0.46-1.91	1.61	0.67-3.85
Waist circumference	1.01	0.99-1.02	1.02	1.001-1.033⁎
Low HDL	1.96	1.15-3.33⁎	1.89	0.89-4.02
Diabetes	1.06	0.53-2.14	1.5	0.77-2.93
Mean SBP	0.988	0.98-1.01	0.99	0.974-1.006
Poverty index <1.30§	2.15	1.03-4.49⁎	1.45	0.61-3.43
Poverty index 1.30 - ≤1.85§	1.03	0.44-2.41	1.05	0.46-2.41
Education >high school¶	0.79	0.45-1.40	0.39	0.20-0.79⁎
Education high school¶	0.4	0.21-0.77⁎	0.61	0.31-1.22
Smoking status current║	1.16	0.66-2.03	3.67	1.76-7.63⁎
Smoking status former║	2.17	1.13-4.17⁎	2.77	1.19-6.47⁎
History of myocardial infarction	0.2	0.08-0.50⁎	0.26	0.12-0.59⁎
History of heart failure	1.12	0.42-2.95	0.62	0.26-1.44
History of stroke	0.37	0.19-0.74⁎	0.48	0.19-1.24
COPD	2.41	1.27-4.59⁎	1.79	0.88-3.66
Cancer	1.34	0.69-2.62	1.28	0.35-4.64

CI = confidence interval.

⁎ Ratio is significant at 0.05 level.

† Vs 7-8 hours of daily sleep.

‡ Vs Non-Hispanic Whites

§ Vs Poverty index >1.85.

¶ Vs less than high-school.

║ Vs never smoker.

After adjusting for age and gender it was also found that the odds of atypical Angina symptoms in participants with sleep < 7 hours continued to be higher (1.77 [1.002-3.122]) than those getting adequate 7-8 hours of daily sleep. Given the lack of significance of stratified Grade 1 angina at the univariate level, no adjusted analysis was conducted for odds of grade 1 angina.

### 3.4 Sensitivity Analysis

We recategorized sleep duration to <6, 6-9, >9 hours based on the meta-analysis done by M. Jike et al.^30^ and conducted identical analyses which confirmed the robustness of the results (data available as supplementary material*). All analyzes accounted for the complex survey design using poststratification weights and clustering. Statistical analyses were performed using SAS 9.4 (Cary, NC). Statistical significance was defined as *P* < .05. The study population's baseline characteristics showed no significant differences, maintaining consistency across univariate, multivariate, and subgroup analyses thereby reinforcing the findings of the primary analysis.

## 4 Discussion

The prevalence of sleep disorders has been rising significantly, particularly in developed countries like the United States.^1^^,^^5^^,^^6^ To our knowledge, this study is the first to analyze the potential relationship between angina characteristics and daily sleep duration. Our findings reveal a notable association between sleep duration and the likelihood of experiencing Grade 2 angina. Both males and females sleeping >8 hours had more than 2x the odds of Grade 2 angina even after adjusting for covariates. Individuals with <7 hours of daily sleep had elevated unadjusted odds of Grade 2 angina, but lost significance after adjusting for confounders. Our analysis also showed that <7 hours of daily sleep was linked to an increased likelihood of experiencing atypical pain, with an odds ratio of 1.77. However, no significant association was found for longer sleep durations after adjustment.

These findings highlight the complex relationship between sleep patterns and angina, indicating that both short (<7 Hours/day) and long (>8 Hours/day) sleep durations contribute to increased angina risk. Sub-optimal sleep is well-documented to increase the risk of conditions like diabetes,^2^ hypertension,^14^ obesity,^15^ metabolic syndrome.^16^ When sleep is compromised, it can trigger a cascade of health issues, including inflammation^2^ and depression,^17^ all of which can contribute to cardiovascular problems like coronary artery disease, heart failure, and arrhythmias.^3^

The specific impact of suboptimal sleep on angina characteristics remains less clearly defined, though several plausible mechanisms have been proposed. One key pathway involves the body's stress response, which is amplified by elevated morning cortisol levels.^1^^,^^18^ Chronic sleep deprivation may disrupt the autonomic balance, favoring heightened sympathetic activity and reduced parasympathetic tone.^19^ This shift can lead to vasoconstriction, elevated heart rate, and increased myocardial oxygen demand, all of which may exacerbate angina symptoms. Additionally, increased sympathetic activity is often accompanied by elevated catecholamines—particularly norepinephrine—which can stimulate the production of inflammatory mediators such as interleukin-6 (IL-6)^28^ and tumor necrosis factor-alpha (TNF-α).^29^

Suboptimal sleep is also linked to systemic inflammation, with elevated levels of inflammatory markers like TNF-α,^7^ IL-6^8^, and high-sensitivity C-reactive protein (hs-CRP),^9^ which can accelerate the development of atherosclerosis. This pro-inflammatory environment, compounded by autonomic dysregulation, may increase coronary artery narrowing and further contribute to angina. Moreover, alterations in lipid profiles have been observed in individuals with sub-optimal sleep,^20^ potentially heightening the risk of coronary heart disease and angina.^21^

Another factor to consider is the activation of the hypothalamic-pituitary-adrenocortical (HPA) axis and changes in cortisol levels, which can influence cardiovascular function.^22^ Increased cortisol levels may lead to higher blood pressure and heart rate, contributing to the development and severity of angina.

Existing literature supports the association between suboptimal sleep duration and cardiovascular risks. Krittanawong et al.^23^ found that deviations from optimal sleep duration were linked to higher rates of stroke, heart failure, diabetes, and hyperlipidemia. These findings align with our results, reinforcing the notion that both short (<7 Hours/day) and long (>8 Hours/day) sleep durations can be detrimental to cardiovascular health, particularly in relation to angina.

Another study done by Cui et al.^24^ found that the link between long (>9 Hours/day) sleep duration and cardiovascular disease (CVD) risk was especially pronounced in individuals aged 50 and older. In this demographic, those with chronic conditions such as hypertension, diabetes, and obesity faced a significantly higher risk of CVD with longer sleep durations. This suggests that increased arterial stiffness, greater blood pressure variability, and underlying diabetes may contribute to these findings, providing a potential explanation for the association between long sleep duration and angina in this age group.

The association between long sleep duration and adverse outcomes remains debated, with evidence suggesting it may reflect underlying illness rather than a direct causal relationship—raising the possibility of ascertainment bias (Grandner et al.).^30^ While our study was not designed to address this question directly, it remains an important consideration in interpreting these findings.

Our study had several strengths, including nationally representative sample, assessment of confounders, and validated angina questionnaire to reduce misclassification bias. However, it has several limitations. First, it was limited in power to fully assess the relationship between sleep duration and angina. The cross-sectional design restricts causal interpretation. Although we adjusted for multiple confounders determined a priori, residual confounding is likely. Sleep duration was assessed using self-reported data, which may introduce recall bias; additionally, sleep quality was not assessed, and future studies should incorporate both objective sleep measures and evaluations of sleep quality. Additionally, psychological factors such as anxiety and depression were not accounted for due to data unavailability. Lastly, survivor bias is possible, as a history of myocardial infarction or stroke appeared to be strongly protective in our findings.

In conclusion, our study highlights the complex interplay between sleep duration and angina characteristics, emphasizing the need for further research to elucidate the underlying mechanisms. Understanding how sleep duration affects angina specifically, rather than just cardiovascular health in general, is crucial for developing targeted interventions that can optimize sleep and reduce the risk of angina in vulnerable populations.

## 5 Conclusion

Our findings indicate that individuals sleeping more than 8 hours are two to three times more likely to experience Grade 2 angina, while those sleeping less than 7 hours are more likely to report atypical symptoms, potentially complicating diagnosis. This underscores the need to consider sleep patterns in angina management. Clinicians may incorporate brief assessments—such as the ROSE questionnaire^13^ for angina symptoms and simple questions on sleep duration and quality—to identify potential sleep-related factors. However, further research is needed to confirm these associations and inform clinical guidelines.

## CRediT authorship contribution statement

**Maslahuddin HA Alhaque Roomi:** Writing – review & editing, Writing – original draft, Resources, Project administration, Methodology, Investigation, Formal analysis, Conceptualization. **Nehal Eid:** Writing – review & editing, Writing – original draft, Resources, Project administration, Methodology, Investigation, Formal analysis, Conceptualization. **Aayush Visaria:** Writing – review & editing, Visualization, Validation, Supervision, Software, Investigation, Formal analysis, Data curation.

## Declaration of competing interest

None.

## Appendix Supplementary materials

Image, application 1
