# Supplementary material for: Association Between Sleep Duration and Angina Characteristics in United States Adults
Source: Am J Med Open. 2025 Jun 19;14:100109. doi: 10.1016/j.ajmo.2025.100109 (PMC12282262; doi:10.1016/j.ajmo.2025.100109)
Supplement: Supplementary file 1 [file mmc1.docx]

| 1. **Sleep Hours Stratification of Study Population** | | |
| --- | --- | --- |
| **Sleep Hours (Rounded)** | **Percent %** | **Frequency** |
| 1 | 0.01 | 6 |
| 2 | 0.23 | 55 |
| 3 | 0.64 | 171 |
| 4 | 3.03 | 699 |
| 5 | 6.58 | 1460 |
| 6 | 18.43 | 3546 |
| 7 | 27.93 | 4661 |
| 8 | 28.87 | 5008 |
| 9 | 9.71 | 1732 |
| 10 | 3.14 | 657 |
| 11 | 0.73 | 177 |
| 12 | 0.54 | 126 |
| 13 | 0.03 | 16 |
| 14 | 0.05 | 8 |
| 15 | 0.001 | 1 |
| Total | 100 | 18323 |

| 1. **Sensitivity Analysis Data: Sleep Hours <6 Hours and >9 Hours v.s 6-9 Hours** | | | | |
| --- | --- | --- | --- | --- |
| **Odds of Grade 2 Angina** | | | | |
| a. | **Males** | **Daily Sleep** | **OR** | **95% CI** |
|  |  | **<6 Hours** | 4.004 | 1.92-8.33* |
|  |  | **>9 Hours** | 2.98 | 1.23-7.22* |
|  | **Females** | **<6 Hours** | 2.44 | 1.29-4.62* |
|  |  | **>9 Hours** | 2.29 | 1.08-4.86* |
| **Odds of Grade 1 Angina** | | | | |
| b. | **Males** | **<6 Hours** | 1.52 | 0.93-2.47 |
|  |  | **>9 Hours** | 1.53 | 0.81-2.88 |
|  | **Females** | **<6 Hours** | 1.31 | 0.84-2.03 |
|  |  | **>9 Hours** | 2.02 | 1.14-3.57* |
| **Ratio is significant at 0.05 level. Abbreviations: OR = Odds Ratio, CI = Confidence Interval.*   1. *Odds of Grade 2 Angina for different daily sleep categories as compared with 6-9 hours of daily sleep* 2. *Odds of Grade 1 Angina for different daily sleep categories as compared with 6-9 hours of daily sleep* | | | | |
